# Unsaturated Copolyesters from Macrolactone/Norbornene: Toward Reaction Kinetics of Metathesis Copolymerization Using Ruthenium Carbene Catalysts

**DOI:** 10.3390/ijms23094521

**Published:** 2022-04-20

**Authors:** Araceli Martínez, Daniel Zárate-Saldaña, Joel Vargas, Arlette A. Santiago

**Affiliations:** 1 Escuela Nacional de Estudios Superiores, Unidad Morelia, Universidad Nacional Autónoma de México, Antigua Carretera a Pátzcuaro No. 8701, Col. Ex. Hacienda de San José de la Huerta, Morelia C.P. 58190, Michoacán, Mexico; danfacquim@hotmail.com (D.Z.-S.); arlette_santiago@enesmorelia.unam.mx (A.A.S.); 2 Instituto de Investigaciones en Materiales, Unidad Morelia, Universidad Nacional Autónoma de México, Antigua Carretera a Pátzcuaro No. 8701, Col. Ex. Hacienda de San José de la Huerta, Morelia C.P. 58190, Michoacán, Mexico; jvargas@iim.unam.mx

**Keywords:** ROMP, macrolactones, norbornene, copolyesters, ruthenium-carbene

## Abstract

Unsaturated copolyesters are of great interest in polymer science due to their broad potential applications and sustainability. Copolyesters were synthesized from the ring-opening metathesis copolymerization of *ω*-6-hexadecenlactone (HDL) and norbornene (NB) using ruthenium-alkylidene [Ru(Cl_2_)(=CHPh)(1,3-*bis*(2,4,6-trimethylphenyl)-2-imidazolidinylidene)(PCy_3_)] (**Ru1**), [Ru(Cl)_2_(=CHPh)(PCy_3_)_2_] (**Ru2**), and ruthenium-vinylidene [RuCl_2_(=C=CH(*p*-C_6_H_4_CF_3_))(PCy_3_)_2_] (**Ru3**) catalysts, respectively, yielding HDL-NB copolymers with different ratios of the monomer HDL in the feed. The activity of *N*-heterocyclic-carbene (NHC) (**Ru1**) and phosphine (**Ru2** and **Ru3**) ligands containing ruthenium-carbene catalysts were evaluated in the synthesis of copolymer HDL-NB. The catalysts **Ru1** with an NHC ligand showed superior activity and stability over catalysts **Ru2** and **Ru3** bearing PCy_3_ ligands. The incorporation of the monomers in the copolymers determined by ^1^H-NMR spectroscopy was similar to that of the HDL-NB values in the feed. Experiments, at distinct monomer molar ratios, were carried out using the catalysts **Ru1**–**Ru3** to determine the copolymerization reactivity constants by applying the *Mayo–Lewis* and *Fineman–Ross* methods. The copolymer distribution under equilibrium conditions was studied by the ^13^C NMR spectra, indicating that the copolymer HDL-NB is a gradient copolymer. The main factor determining the decrease in melting temperature is the inclusion of norbornene units, indicating that the PNB units permeate trough the HDL chains. The copolymers with different molar ratios [HDL]/[NB] have good thermal stability up to 411 °C in comparison with the homopolymer PHDL (384 °C). Further, the stress–strain measurements in tension for these copolymers depicted the appreciable increment in stress values as the NB content increases.

## 1. Introduction

Aliphatic polyesters derived from renewable feedstock have drawn much attention for a long time due to their wide potential applications and sustainability. The macrolactones such as ambrettolide, *ω*-6-hexadecenlactone (6HDL), pentadecalactone (PDL), or ethylene brassylate (EB) can be isolated from plant oils [1], and they can also be obtained from the full fatty acid chain via self-metathesis [2,3]. A high versatility in the production of homopolymers and various block and random macrolactone-based copolymers with cyclic esters has been afforded by ring-opening polymerization (ROP) using aluminum salen complexes [4,5,6,7,8] and enzymatic catalysts [9,10,11,12]. Another method by which bio-based polyesters are obtained effectively is from ring-opening metathesis polymerization (ROMP) [13,14,15,16,17]. The catalytic ROMP of unsaturated macrolactones to yield polymacrolactones using ruthenium-alkylidene (first- and second-generation Grubbs catalysts) and ruthenium vinylidene catalysts has great potential since this route allows the formation of high molecular weight products with excellent mechanical and thermal properties, which has led to their use in a wide range of applications, from commodity-type to engineering materials. For example, the synthesis of *ω*-6-hexadecenlactone to obtain unsaturated linear poly(*ω*-6-hexadecenlactone) with remarkable thermal, mechanical, and biodegradability properties was reported [13]. Other researchers studied the one-pot combination of enzymatic ring-opening polymerization (eROP) and ROMP to prepare block copolymers from cyclooctadiene with lactones using Novozym 435 and Grubbs second-generation catalysts [14]. Moreover, the combination of unsaturated macrolactone (i.e., ambrettolide (Amb)) and the cycloolefin *cis*-cyclooctene (*c*CO) to yield aliphatic long-chain polyesters was studied. The large number of methylene units in the backbone allows these polymers to have a molecular structure similar to that of high-density polyethylene (HDPE) [18].

On the other hand, one of the attractive points of ROMP is the use of cycloolefins such as norbornene and its functionalized derivatives (since norbornene is relatively cheap and industrially available); in addition, most of the norbornene derivatives have exhibited high activity towards ROMP, making them a point of study over the last two decades [19,20]. Besides the design, theoretical, and experimental studies of homopolymers obtained via ROMP using norbornene derivatives, the copolymerization of these monomers has been studied because this polymerization technique has a high tolerance towards many functional groups and oxygen, and this method can proceed under mild reaction conditions at high polymerization rates, especially with strained cyclic olefins [21]. Several studies have focused on obtaining block copolymers via ROMP using those strained cycloolefins [22,23,24,25], but there are only a few reports on the obtaining random precision copolymers, due to the strict selection of the catalyst, and mostly because of the differences in reactivity in the monomers used that impact the same incorporation of both fragments [26,27,28,29].

A limited example of this is the incorporation of a macrocycle fragment into a perfect alternating copolymer architecture with a norbornene derivative fragment because two parameters could have difficulty with this incorporation; first, the symmetry of the macrocycle to the vicinal carbons of the olefin brings several ways of accommodating the fragment, and second, the polymerization and homogenous incorporation of the macrocycle fragment via ROMP to the final material. For instance, based on the ROMP of cycloolefins, it has been reported that the copolymerization of norbornene and cyclooctene by cross-metathesis of polynorbornene with polyoctenamer using first-generation Grubbs catalyst exhibits low activity toward copolymerization of those monomers [30]. Even, the polymerization of macrocycles is a wide field of study, receiving a special name of entropy-driven ROMP (ED-ROMP) since this polymerization is not based on the strain of the olefin monomer but only on the entropic changes from cyclic to linear fragment [31,32,33,34]. With the homogenous incorporation of a macrocycle though the copolymeric material, very interesting hydrolysable bonds could be achieved for biodegradation and optimization of crystallinity, promising electro-optical applications, among others [18,32,35]. In this context, and to the best of our knowledge, we report for the first time on the copolymerization of *ω*-6-hexadecenlactone and polynorbornene using the ruthenium-alkylidene Ru(Cl_2_)(=CHPh)(1,3-*bis*(2,4,6-trimethylphenyl)-2-imidazolidinylidene)(PCy_3_)] (**Ru1**), [Ru(Cl)_2_(=CHPh)(PCy_3_)_2_] (**Ru2**), and ruthenium-vinylidene [RuCl_2_(=C=CH(*p*-C_6_H_4_CF_3_))(PCy_3_)_2_] (**Ru3**) catalysts, respectively, yielding HDL-NB copolymers with different ratios of the monomer HDL in the feed.

## 2. Results and Discussion

**Scheme 1:**
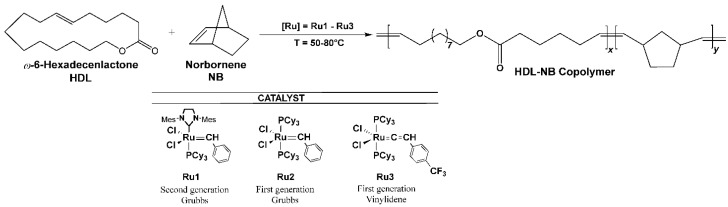
HDL copolymers with NB *via* ROMP.

We have previously reported on the ring-opening metathesis polymerization (ROMP) of homopolymer poly(*ω*-6-hexadecenlactone) (PHDL) from *ω*-6-hexadecenlactone (HDL), an abundant compound in ambrette seed oil (*Hibiscus abelmoschus* L.) [21]. In order to obtain unsaturated polyesters modified with cycloolefins, the *ω*-6-hexadecenlactone (HDL) was copolymerized with norbornene (NB) *via* ROMP. The copolymerizations of HDL-NB were accomplished at 50 °C using ruthenium-alkylidene Grubbs Ru(Cl_2_)(=CHPh)(1,3-*bis*(2,4,6-trimethylphenyl)-2-imidazolidinylidene)(PCy_3_)] (**Ru1**) and [Ru(Cl)_2_(=CHPh)(PCy_3_)_2_] (**Ru2**), and at 80 °C with ruthenium-vinylidene [RuCl_2_(=C=CH(*p*-C_6_H_4_CF_3_))(PCy_3_)_2_] (**Ru3**) catalysts [36] (Scheme 1).

At first, the homopolymerization of HDL and NB was performed, and subsequently, their copolymerization, in which the initial ratio of the two monomers was determined by molar ratio [HDL]/[NB] (Table 1). The maximum yield (96%) of poly(*ω*-6-hexadecenlactone) (PHDL) was achieved in three hours using the **Ru1** catalyst with a molecular weight around *M_n_* = 109,600 g mol^−1^ (*entry 1*). The homopolymerization of NB was completed in less than one hour using **Ru1** catalyst to give poly(norbornene) (PNB) with molecular weight around 134,000 g mol^−1^ and yield of 99% (*entry 2*). Table 1 shows the results of the copolymerization HDL-NB that were synthesized using different ratios of the monomer HDL in the feed. In order to reach full conversion and thermodynamic equilibrium of the HDL-NB copolymers, the reactions were allowed to run for 20 h. The activity of *N*-heterocyclic-carbene (NHC) (**Ru1**) and phosphine (**Ru2 and Ru3**) ligands containing ruthenium-carbene catalysts were evaluated in the synthesis of copolymer HDL-NB. We can observe in Table 1 that the copolymers were successfully synthesized, with yields ranging from 96% to 98% and molecular weight around *M_n_* = 1 × 10^5^ g mol^−1^, when **Ru1** catalyst is used (*entries* *7, 10–12, 15, and 16*). The catalysts **Ru1** with an NHC ligand showed superior activity upon catalysts **Ru2** and **Ru3** bearing PCy_3_ ligands. The copolymers HDL-NB using **Ru2** and **Ru3** catalysts had molecular weight values around *M_n_* = 1 × 10^4^ g mol^−1^, with yields ranging from 53% to 66% and from 42% to 57%, respectively (*entries 8, 9, 13 and 14*). It has been demonstrated that the free Gibbs activation energies of metathesis are consistently higher for catalyst **Ru1** than for **Ru2**, with better transition state stabilization by the IMesH_2_ ligand compared with PCy_3_ [37].

**Figure 1:**
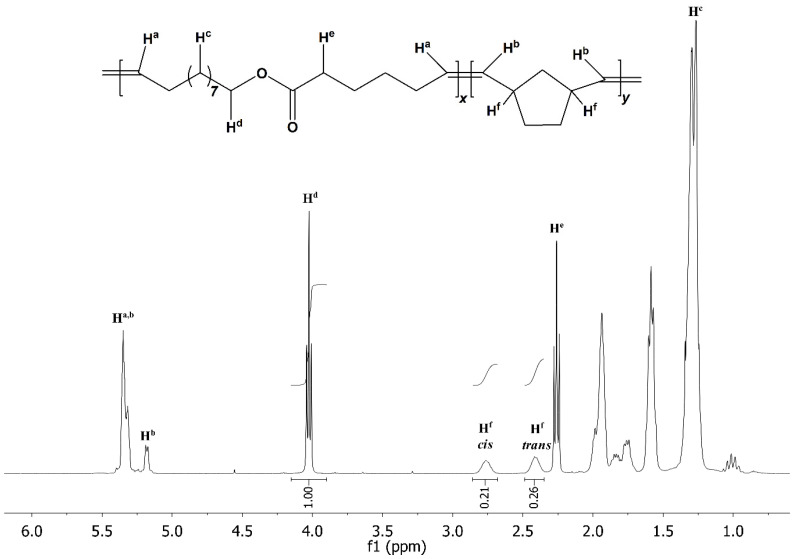
^1^H-NMR (400 MHz, CDCl_3_) spectrum of the copolymer HDL-NB (Table 1, *entry 12*).

The composition of HDL-NB copolymers with several molar ratios determined using ^1^H-NMR analysis is shown in Table 1. It can be seen that the composition of each copolymer obtained by ^1^H-NMR integration was similar according to the theoretical results (*entries 7–16*). Figure 1 shows ^1^H-NMR spectrum of copolymer HDL-NB using a ratio molar ([HDL]/[NB] = 2 and the catalyst **Ru1** (Table 1, *entry 12)*. The incorporation of HDL was obtained by integrating the area of the methylene ester groups region (*δ* = 4.05 ppm, HDL) relative to the CH protons (*δ* = 2.79 (*cis*), 2.44 (*trans*) ppm), NB). From the relative intensities of these signals, the ratio of HDL-NB copolymer was found to be 82/18%. The spectrum also shows the signals arising in the 5.35–5.12 ppm region, which correspond to the protons of the HDL-NB double bonds (=CH).

The copolymerization of HDL-NB was studied as a function of time. Copolymerization was synthesized using a molar ratio [HDL]/[NB] = 1 at 50 °C with **Ru1** catalyst (Table 1, *entries* 3–7). The HDL incorporation percentage in PNB was determined by ^1^H-NMR. We can see from Table 1 that during copolymerization, NB monomer was polymerized faster than HDL due to its high cyclic strain. HDL incorporation in NB was 39–42% over the period from 4 to 30 min (*entries 3 and 4*). In this time, the molecular weight of the copolymer was increased four orders of magnitude *M_n_* × 10^4^ g mol^−1^. After 3 h, the HDL incorporation in NB was increased until the copolymerization reached equilibrium HDL-NB with a molecular weight *M_n_* × 10^5^ g mol^−1^ (*entry 7*).

On the other hand, the copolymerizations of HDL and NB via ROMP, for reactivity constants determination, were carried out at 50 °C and 80 °C using **Ru1–Ru3** catalysts (Scheme 1). For each catalyst, the reactions were conducted at 50, 60, 67, 75, and 91 mol percentage of HDL in the feed and polymerized up to a conversion of max. 22.10 (**Ru1**), 19.50 (**Ru2**), and 15.70 (**Ru3**) weight percentage, respectively (Table 2). The molar ratio of C=C monomer to **Ru1** was 500. Since at the conversion desired monomer HDL was not detected in the copolymer formed when the reactions were conducted at a molar ratio of [C=C]/[**Ru2, Ru3**] = 500, the experiments for reactivity constant determination were carried out at 250. The copolymer was separated from the residual monomer by precipitation, and the incorporation of HDL in the different copolymers synthesis was quantified by ^1^H-NMR (*entries 1–15*). Additionally, in Table 2 it can be seen that as the mol percentage of HDL in the feed increased for each catalyst, more reaction time was needed for copolymerization to take place until the conversion desired. The time required for incorporating HDL into the copolymer was relatively short when the catalyst **Ru1** was used (*entries 1–5*), while for the presence of the catalysts **Ru2** and **Ru3**, long periods from 2 to 7 h were required (*entries 6–15*).

On the basis of the results obtained from the copolymerization experiments, *Mayo–Lewis* [38] and *Fineman–Ross* [39] methods were applied to calculate the reactivity constants r_HDL_ and r_NB_ in the presence of **Ru1**–**Ru3** catalysts, using the general copolymerization equation. Table 3 shows the reactivity constants r_HDL_ and r_NB_ obtained by the different methods. By applying the *Mayo–Lewis* method, the values obtained were similar to those values obtained for the *Fineman–-Ross* method. It is important to note that the precision of experimentally determined monomer reactivity ratio depends on the experimental design and technique used to analyze the data [40]. It has been reported that the ring strain energy for norbornene is 27.2 kcal/mol, which is adequately high and therefore can be easily polymerized [41]. In contrast, large cyclic esters—for example, monomers containing 12 or more atoms—are lactones with unstrained rings [42,43]. In this context, the influence of the catalysts on the reactivity of the monomer is of great interest. For example, by applying the *Mayo–Lewis method,* the values obtained were r_HDL_ = 0.24 and r_NB_ = 3.78 when the second-generation Grubbs (**Ru1**) catalyst was used. The reactivity of HDL was changed considerably by alkylidene **Ru2** or vinylidene **Ru3** catalysts (Table 3).

**Figure 2:**
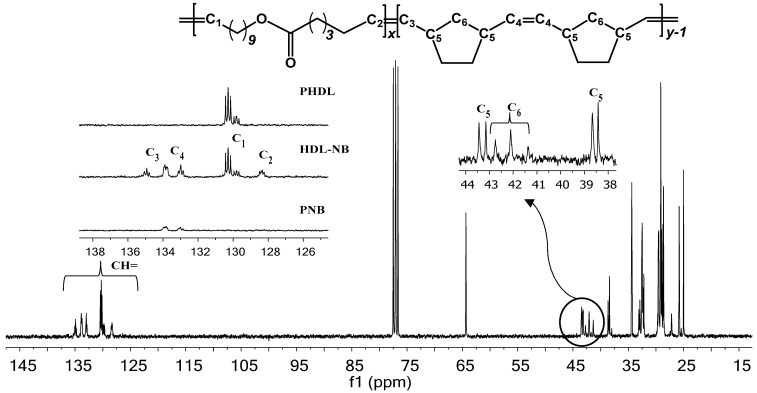
^13^C-NMR (100 MHz, CDCl_3_) spectrum for the copolymer HDL-NB (Table 1, *entry 7*).

The homopolymers distribution of copolymers was studied. The copolymerization HDL-NB was synthesized until reaching full conversion and thermodynamic equilibrium for 20 h. The ^13^C spectrum of copolymer HDL-NB (Figure 2) (Table 1, *entry 7*) shows several extra signals in the double-bound region at 135.05–134.77 ppm and 128.47–128.22 ppm; compared with both homopolymers, these signals may be due to the gradual change in composition from NB to HDL. A significant NB-NB homopolymer section was also observed. The spectrum shows signals at 43.43 and 38.41 ppm, which correspond the C-H of the norbornene. It suggests that the HDL-NB copolymer is a gradient copolymer. We can see that the spectra of the homopolymers have two similar signals observed at 130.40 ppm and 129.60 ppm for poly(*ω*-6-hexadecenlactone (PHDL) and 132.84 ppm and 133.89 ppm for polynorbornene (PNB), which correspond to the double-bond *trans* and *cis* configuration, respectively (Figure 2).

**Figure 3:**
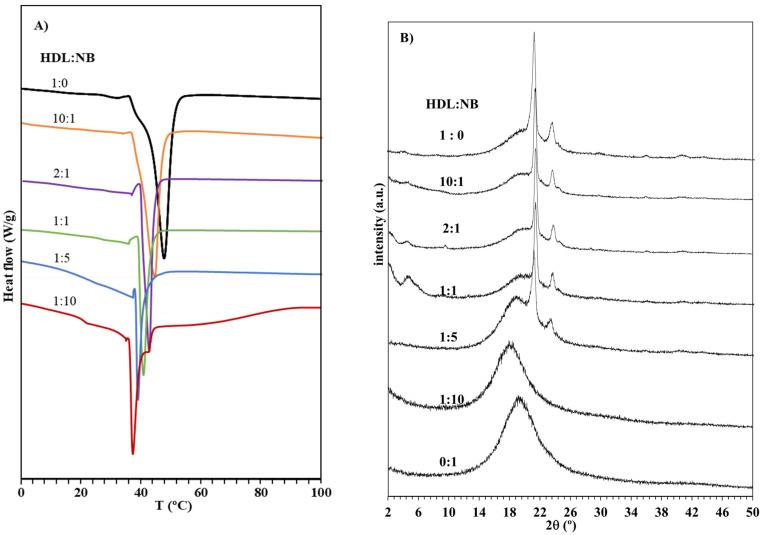
(**A**) Second-heat thermograms of DSC and (**B**) X-ray diffraction pattern of PHD, PNB, and HDL-NB copolymers (from 10:1 to 1:10).

Differential scanning calorimetry (DSC) provides a tool for monitoring the HDL-NB copolymers’ crystallinity. All thermal transition temperatures of the HDL-NB copolymers were determined using second heating scans from −80 °C to 300 °C at 10 °C/min. Figure 3A shows the DSC thermograms of pure PHDL and all HDL-NB copolymers except amorphous PNB. Table 4 contains the melting point T_m_ and enthalpy of fusion ΔH_m_ parameters of the studied copolymers extracted from the DSC data; the glass transition temperature (T_g_) was not detected in the scanned heating range. An endothermic peak was observed around 47.60 °C, as was an enthalpy of fusion ΔH_m_ = 73 J/g, which corresponds to the melting point of PHDL. It is possible to observe that the T_m_ and ΔH_m_ decreased as the norbornene amount is increased, indicating that the PNB units permeate through the HDL chains.

The thermal stability of copolymers was also studied by thermogravimetric analysis (TGA) under N_2_ (Table 4). TGA experiments show that copolymers with different molar ratios [HDL]/[NB] have good thermal stability up to 411 °C, by comparison with the homopolymer PHDL (384 °C). As expected, the copolymers containing a greater percentage of HDL exhibit a little lower decomposition temperature. The latter is attributed to the lower norbornene content in the HDL-NB copolymers (*entries 3–7*). This result indicates that the norbornene segment in the copolymer improved its thermostability relatively.

Figure 3B shows the X-ray diffraction patterns of HDL-NB copolymers determined through X-ray diffraction analysis in the 2θ range of 2–50° (Table 4, *entries 1–7*). Whereas PNB and the HDL-NB (1:10) copolymer are amorphous, the PHDL and the HDL-NB copolymers (10:1, 2:1, 1:1, and 1:5) are semicrystalline. The PHDL X-ray pattern presents a very strong crystalline peak at 2θ of 21° and three weak crystalline peaks at 2θ of 23°, 36°, and 41°. We can observe that the X-ray patterns of HDL-NB copolymers (10:1. 2:1, and 1:1) are similar to that of PHDL, except for the presence of the peaks at 2θ of 4.5°. PHDL displayed the highest crystallinity of 31%, followed by HDL-NB = 10:1 (26.5%,), 2:1 (23.0%), and 1:1 (19.8%) copolymers, respectively (Table 4, *entries 2–5*). For HDL-NB (1:5), the copolymer shows crystalline peaks in 21° and 23° overlapped with a large amorphous halo centered at 2θ of 19°. The percentage crystallinity calculated was 15.9%. The PNB pattern presents one broad peak with a maximum 2θ value of 19.6°, while the pattern of HDL-NB (1:10) presents only a broad amorphous halo with one well-defined hump at 18.2°.

**Figure 4:**
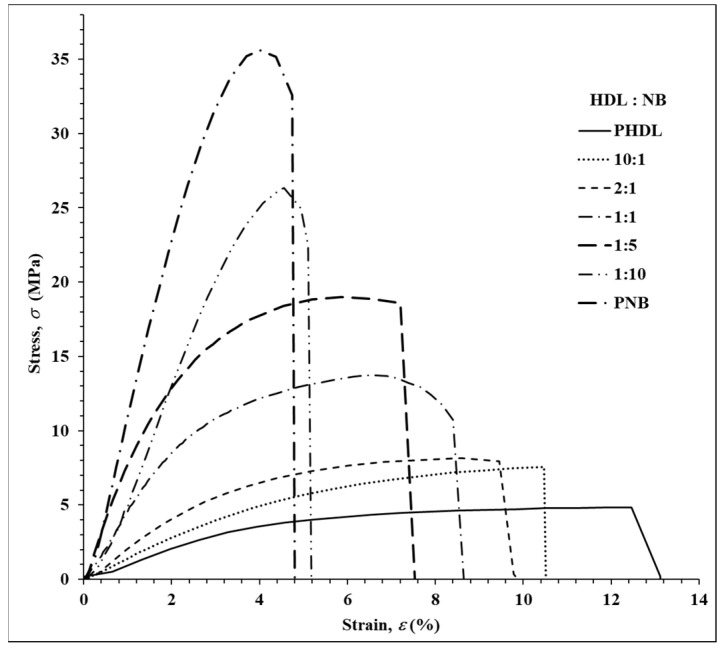
Strain–stress curves of pure PNB, PHDL, and HDL-NB copolymers with several molar ratios [HDL]/[NB].

Stress–strain measurements using the tensile test for the films of the HDL-NB copolymers were studied (Table 4, *entries 3–7*). Figure 4 shows the tensile stress–strain curves depicting an appreciable increment in the stress values as the NB monomer content increases. Each curve was cut at the maximum stress and indicates that not only the stress (*σ* = 7.55 MPa to 26.33 MPa) but also the elastic modulus (*E* = 156 MPa to 775 MPa) for copolymers (Table 4, *entries 3–7*) are higher than those mechanical properties exhibited for PHDL (*σ* = 4.84 MPa and *E* = 119 MPa, respectively) (*entry 2*).

## 3. Materials and Methods

### 3.1. Materials and Characterization Techniques

[Ru(Cl_2_)(=CHPh)(1,3-*bis*(2,4,6-trimethylphenyl)-2-imidazolidinylidene)(PCy_3_)] (second-generation Grubbs catalyst) (**Ru1**), [Ru(Cl)_2_(=CHPh)(PCy_3_)_2_] (first-generation Grubbs catalyst) (**Ru2**), norbornene, *ω*-6-hexadecenlactone (≥98%, HDL), chlorobenzene anhydrous, and methanol were purchased from Aldrich Chemical Co. and used as received. Ru-vinylidene catalyst [RuCl_2_(=C=CH(*p*-C_6_H_4_CF_3_))(PCy_3_)_2_] (**Ru3**) was synthesized following the procedure described in our previous work [36]. 1,2-Dichloroethane was dried over anhydrous calcium chloride and distilled over CaH_2_.

Number-average molecular weight (*M_n_*) and molecular weight distributions (MWD) were determined with reference to monodisperse polystyrene standards on a Waters 2695 ALLIANCE Separation Module GPC at 30 °C in tetrahydrofuran (THF) equipped with a universal column and with a flow rate of 0.3 mL/min. Nuclear magnetic resonance (NMR) spectra were recorded at 298 K with a Bruker AVANCE 400 MHz spectrometer, at 400 MHz (^1^H) and 100 MHz (^13^C). The chemical shifts are provided in parts per million from SiMe_4_ (^1^H and ^13^C) as internal reference. The samples (100 mg) were mixed in 3.30 mL of CDCl_3_. The melting point (T_m_) and enthalpy of fusion (ΔH_m_) of the HDL-NB copolymers samples were measured by differential scanning calorimetry (DSC) analysis using a TA Instrument Q20 with a heat-rate of 10 °C/min under a nitrogen flow of 50 mL/min in the range of −80 °C to 300 °C. The decomposition onset temperature, T_d_, was determined using thermogravimetric analysis, TGA, using a heat rate of 10 °C/min under nitrogen atmosphere with a DuPont 2100 instrument. X-ray diffraction measurements of HDL-NB copolymers films were carried out in a Siemens D-5000 diffractometer between 2 and 50 degrees 2θ, at 35 KV 25 mA, using CuK_α_ radiation (1.54 Å). Mechanical properties under tension, elastic modulus (*E*), stress (*σ*), and strain (*ε*) were measured in a Universal Mechanical Testing Machine Instron 1125–5500 R using a 50 Kg cell at a crosshead speed of 10 mm/min according to the method ASTM D1708 in film samples of 0.5 mm of thickness at room temperature.

### 3.2. General Monomer Polymerization

The ROMP reactions of NB and HDL to obtain their corresponding homopolymers and copolymers were carried out in glass vials under nitrogen atmosphere. The polymerizations were inhibited by adding a small amount of ethyl vinyl ether, and the resulting solution of the reaction was poured into an excess of methanol. The resulting homopolymers and copolymers were purified by solubilization in chloroform and further precipitation into methanol. The fibrous products were dried in a vacuum oven at 40 °C to constant weight.

#### 3.2.1. Synthesis of Polynorbornene (PNB)

The general polymerization was followed, dissolving 1 g (10.62 mmol) of norbornene (NB) in 10.60 mL of 1,2-dichloroethane at room temperature. Then, 8.74 mg (0.0106 mmol) of **Ru2** catalyst was added, and the mixture was stirred for 40 min. Yield = 99 %. ^1^H NMR (*400 MHz, CDCl_3_, ppm*): δ 5.35 (*s*, CH=CH *trans*); δ 5.21, 5.19 (*d*, CH=CH *cis*); δ 2.79 (*bs*, *s*, CH); δ 2.44 (*bs*, *trans*, CH_2_); δ 1.87–1.76 (*m*, CH_2_); δ 1.55 (*s*, CH_2_); δ1.11–0.98 (*m*, CH_2_). ^13^C NMR (*100 MHz, CDCl_3_, ppm*): δ 133.98 (*d*, C=C); δ 41.34, 33.89, 32.18 (*s, d, d*, CH_2_-); δ 43.13, 38.65 (*d, d*, C=CH_2_*, trans, cis*).

#### 3.2.2. Synthesis of Poly(*ω*-6-Hexadecenlactone) (PHDL)

The general polymerization technique previously described was followed, dissolving 1 g (3.96 mmol) of *ω*-hexadecenlactone (HDL) in 4.00 mL of 1,2-dichloroethane. Afterwards, 6.52 mg (0.008 mmol) of **Ru1** catalyst was added. The reaction mixture was maintained at 50 °C for 3 h. Yield = 96 %. ^1^H NMR (*400 MHz, CDCl_3_, ppm*): δ 5.37, 5.21 (*bs*, *trans*, -CH=CH-); δ 4.05 (*t*, CH_2_-O); δ 2.26 (*t*, CH_2_-CO_2_); 1.94–1.98 (*m*, CH_2_-C=); 1.60–1.56, 1.30–1.26 (*m*, CH_2_-). ^13^C NMR (*100 MHz, CDCl_3_, ppm*): δ 130.28, 129.83 (*t*, *t*, C=C); 64.36 (*s*, CH_2_-O); δ 34.37 (s, CH_2_-CO); 32.54 (s, C=CH_2_); 29.58–28.58, 25.77, 24.98 (m, −CH_2_).

#### 3.2.3. Synthesis of HDL-NB Copolymers

The synthesis route of the copolymer is shown in Scheme 1. The HDL-NB copolymerization was carried out in a glass vial under dry nitrogen atmosphere. *ω*-6-Hexadecenlactone monomer (HDL) (2.00 g, 7.92 mmol) and norbornene monomer (NB) (0.75 g, 7.92 mmol)) were initially dissolved in 1,2-dichloroethane (1 mol/L) at a ratio molar [HDL]/[NB] = 1. The HDL-NB copolymerizations were synthesized using a different ratio of the monomer HDL in the feed ([HDL]/[NB] = 1:1, 1:5, 1:10, 2:1, 3:1, and 10:1). Then, catalyst **Ru1** (1.30 × 10^−2^ g, 1.58 × 10^−2^ mmol) was added, and the mixture was stirred at 50 °C. When the **Ru2** catalyst was used, the reaction temperature was set at 80 °C. HDL-NB copolymer was synthesized using different molar ratios [HDL-NB]/[**Ru1–Ru3**] = 500, 250. After being inhibited by adding a small amount of ethyl vinyl ether (0.3 mL, 3.00 mmol), the solution was poured into an excess of methanol. The copolymer was purified by solubilization in chloroform containing a few drops of 1 N HCl and precipitation into methanol. The obtained polymer was dried in a vacuum oven at 40 °C to constant weight. **^1^H NMR (*400 MHz, CDCl_3_, ppm*)**: δ 5.37, 5.21 (*bs*, CH=CH); δ 4.05 (*t*, CH_2_-O); δ 2.79 (*bs*, *cis*, CH_2_-C=, NB); δ 2.44 (*bs*, *trans*, CH_2_-C=, NB); δ 2.28 (*t*, CO-CH_2_); δ 1.96 (*bs*, CH_2_-COO-); 1.94–1.77, 1.32, 1.11–0.98 (*m*, *bs*, *m*, CH_2_, NB); δ 1.61, 1.32 (*m*, *bs*, -CH_2_, HDL); δ 1.61 (*m*, =C-CH_2_, HDL). **^13^C NMR (*100 MHz, CDCl_3_, ppm*):** δ 173.93 (*s*, -C=O); δ 135.34–128.05 (*m*, C=C); δ 64.35 (*s*, CH_2_-O); δ 43.13, 38.65 (*d, d*, C=CH_2_*, trans, cis*); δ 34.37 (*s*, CH_2_-CO); δ 41.34, 33.89, 32.18 (*s, d, d*, CH_2_-, NB); δ 32.57, 27.19 (*d, d* = C-CH_2_, HDL*, trans, cis*); δ 29.57–28.63, 25.80, 25.01 (*m, s, s*, -CH_2_, HDL).

## 4. Conclusions

The ruthenium-alkylidene (**Ru1** and **Ru2**) and ruthenium-vinylidene (**Ru3**) catalysts showed high catalytic efficiency in the ring-opening of the *ω*-hexadecenlactone and norbornene to obtain unsaturated copolyesters. The catalysts **Ru1** with an NHC ligand showed superior activity and stability over catalysts **Ru2** and **Ru3** bearing PCy_3_ ligands. The HDL-NB copolymers were successfully synthesized with yields ranging from 96% to 98% and molecular weights around *M_n_* = 1 × 10^5^ g mol^−1^ when **Ru1** catalyst was used, while the presence of the catalysts **Ru2** and **Ru3** afforded molecular weight values around *M_n_* = 1 × 10^4^ g mol^−1^, with yields ranging from 53% to 66% and from 42% to 57%, respectively. The incorporation of HDL in the NB chain was similar according to the theoretical results when these catalysts are used. The copolymer distribution under equilibrium conditions and considering the long reaction time indicated that the copolymer HDL-NB is a gradient copolymer. The monomer reactivity ratios using catalysts **Ru1**–**Ru3** by applying the Mayo–Lewis and Fineman–Ross methods was studied. The values obtained by both methods were similar r_HDL_ = 0.24, 0.28 and r_NB_ = 3.78, 4.02 when the second-generation Grubbs (**Ru1**) catalyst was used, respectively. The reactivity of HDL was considerably affected by the alkylidene **Ru2** or vinylidene **Ru3** catalysts. The thermal stability of HDL-NB copolymers with different molar ratios was found to be up to 411 °C by comparison with the homopolymer PHDL, and it was observed that the T_m_ and ΔH_m_ decreased as the norbornene amount was increased, indicating that the PNB units permeate through the HDL chains, showing a distribution of the amorphous and crystalline region. The HDL-NB copolymers obtained by **Ru1-Ru3** catalysts showed improved mechanical properties.

## Figures and Tables

**Table 1:** Copolymerization *via* ROMP of *w*-6-hexadecenlactone (HDL) with norbornene (NB) using **Ru1–3** catalysts.

Entry ^a^	Molar Ratio ^b^		[Ru]	[C=C] ^c^ [Ru]	Time (h)	Temp (°C)	Yield (%) ^d^	*M_n_*^e^ (g mol^−1^)	MWD ^e^	HDL/NB Expected *wt. %*	HDL/NB Measured ^f^ *wt. %*
	HDL NB										
**1**	1	0	**Ru1**	500	3	50	96	109,600	1.15	--	--
**2**	0	1	**Ru1**	1000	40 min	25	99	134,000	1.10	--	--
**3**	1	1	**Ru1**	500	4 min	50	22	78,876	2.80	--	39/61
**4**	1	1	**Ru1**	500	30 min	50	36	81,500	2.90	--	42/58
**5**	1	1	**Ru1**	500	3	50	49	88,677	2.78	--	57/43
**6**	1	1	**Ru1**	500	8	50	72	91,981	2.84	--	61/39
**7**	1	1	**Ru1**	500	20	50	97	104,424	2.10	74/26	72/28
**8**	1	1	**Ru2**	250	20	50	66	94,210	2.00	74/26	70/30
**9**	1	1	**Ru3**	250	20	80	57	92,005	2.09	74/26	69/31
**10**	1	5	**Ru1**	500	20	50	96	110,500	2.20	35/65	32/68
**11**	1	10	**Ru1**	500	20	50	96	118,080	2.00	20/80	17/83
**12**	2	1	**Ru1**	500	20	50	98	114,600	2.10	84/16	82/18
**13**	2	1	**Ru2**	250	20	50	53	90,600	2.10	84/16	79/21
**14**	2	1	**Ru3**	250	20	80	42	88,500	2.38	84/16	77/23
**15**	3	1	**Ru1**	500	20	50	97	115,000	2.10	89/11	87/13
**16**	10	1	**Ru1**	500	20	50	98	113,790	2.00	91/9	89/11

^a^ initial HDL-NB monomers concentration [M_o_] = 1 mol/L. 1,2-Dichloroethane (*entries 1–8, 10–13, 15 and 16*) and chlorobenzene (*entries 9 and 14*) were used as solvents. ^b^ Molar ratio [HDL]/[NB]. ^c^ Molar ratio of [C=C] to catalyst. ^d^ Isolated yield of products. ^e^ The number-average molecular weight (*M_n_*) and molecular weight distribution (MWD) of the polymers and copolymers were calculated by gel permeation chromatography (GPC) with THF as the eluent; values are reported relative to PS standards. ^f^ Determined by ^1^H NMR.

**Table 2:** Copolymerization conditions of HDL and NB monomers for reactivity constants determination.

Entry	Mass of HDL in the Feed (g)	[HDL] ^a^ [NB]	Mol % of HDL in the Feed ^b^	Incorporation of HDL in Copolymer (%) ^c^	Time (h)	Yield ^d^ (%)
**Second-Generation Grubbs (Ru1)**						
**1**	0.48	1:1	50	39.00	4 min	22.10
**2**	0.48	1.5:1	60	48.00	4 min	19.60
**3**	0.48	2:1	67	62.00	6 min	16.50
**4**	0.48	3:1	75	74.00	6 min	8.40
**5**	0.48	10:1	91	86.00	10 min	5.30
**First-Generation Grubbs (Ru2)**						
**6**	0.48	1:1	50	35.40	2	19.50
**7**	0.48	1.5:1	60	42.90	2	15.40
**8**	0.48	2:1	67	48.00	4	13.40
**9**	0.48	3:1	75	61.30	4	10.20
**10**	0.48	10:1	91	78.90	7	7.60
**First-Generation Vinylidene (Ru3)**						
**11**	0.48	1:1	50	27.50	2	15.70
**12**	0.48	1.5:1	60	43.40	2	13.30
**13**	0.48	2:1	67	47.10	4	12.30
**14**	0.48	3:1	75	59.40	4	10.10
**15**	0.48	10:1	91	77.60	7	7.20

^a^ molar ratio [HDL]/[NB]. ^b^ Molar ratio of [C=C] to catalyst = 500, T = 50 °C (*entries* 1–5) and 250, T = 80 °C (*entries* 6–15), initial HDL-NB monomers concentration [M_o_] = 1 mol/L. ^c^ Determined by ^1^H-NMR. ^d^ Methanol insoluble polymer.

**Table 3:** Monomer reactivity constants calculated by using *Mayo–Lewis* and *Finemann–Ross* methods.

	*Mayo–Lewis* Method		*Finemann–Ross* Method	
Catalyst	r_HDA_	r_NB_	r_HDA_	r_NB_
** 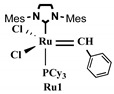 **	0.10	5.60	0.12	5.81
** 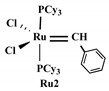 **	0.24	3.78	0.28	4.02
** 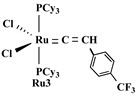 **	0.06	4.47	0.07	4.30

**Table 4:** Thermal and mechanical properties of the copolymerization *via* ROMP of *ω*-6-hexadecenlactone (HDL) with norbornene (NB).

Entry	Molar Ratio ^a^		Thermal Properties			Crystallinity X-ray ^e^	Mechanical Properties ^f^		
	HDL	NB	T_m_^b^ (°C)	T_d_^c^ (°C)	ΔH_m_^d^ (J/g)	(%)	*E* (MPa)	*σ* (MPa)	*ε* (%)
**1**	0	1	--	418	--	--	1280	35.60	4.00
**2**	1	0	47.60	384	73.00	31.00	119	4.84	12.50
**3**	10	1	44.34	390	53.50	26.50	156	7.55	10.47
**4**	2	1	42.10	397	47.10	23.00	229	8.13	8.637
**5**	1	1	40.50	400	43.30	19.80	464	13.73	6.40
**6**	1	5	38.10	409	23.20	15.90	695	19.01	5.87
**7**	1	10	37.20	411	12.00	--	775	26.33	4.55

^a^ Molar ratio [HDL]/[NB], initial HDL-NB monomers concentration [M_o_] = 1 mol/L in 1,2-dichloroethane, and a molar ratio [C=C]/[Ru1] = 500. ^b^ The peak of the melting exotherm in DSC. ^c^ Reported as the onset of 5% decomposition recorded by TGA at a heating rate of 20 °C/min in N_2_. ^d^ Enthalpy of fusion determined by DSC. ^e^ Calculated from X-ray. ^f^ Mechanical properties under tension, elastic module (*E*), stress (*σ*), and strain (*ε*).

## Data Availability

The data presented in this study are available on request from the corresponding author.

## References

[1] Pepels M.P.F., Govaert L.E., Duchateau R. (2015). Influence of the Main-Chain Configuration on the Mechanical Properties of Linear Aliphatic Polyesters. Macromolecules.

[2] Stempfle F., Ortmann P., Mecking S. (2013). Which polyesters can mimic polyethylene?. Macromol. Rapid Commun..

[3] Ogawa R., Hillmyer M.A. (2021). High molar mass poly(ricinoleic acid): Via entropy-driven ring-opening metathesis polymerization. Polym. Chem..

[4] Pepels M.P.F., Bouyahyi M., Heise A., Duchateau R. (2012). Kinetic investigation on the catalytic ring-opening (co)polymerization of (macro)lactones using aluminum salen catalystsIt. Macromolecules.

[5] Fuoco T., Meduri A., Lamberti M., Venditto V., Pellecchia C., Pappalardo D. (2015). Ring-opening polymerization of ω-6-hexadecenlactone by a salicylaldiminato aluminum complex: A route to semicrystalline and functional poly(ester)s. Polym. Chem..

[6] Gong S., Du P., Ma H. (2018). Binuclear aluminum complexes supported by linked bis(*β*-diketiminate) ligands for ring-opening polymerization of cyclic esters. Chin. J. Polym. Sci..

[7] Bouyahyi M., Duchateau R. (2014). Metal-Based Catalysts for Controlled Ring-Opening Polymerization of Macrolactones: High Molecular Weight and Well-Defined Copolymer Architectures. Macromolecules.

[8] D’Auria I., Santulli F., Ciccone F., Giannattasio A., Mazzeo M., Pappalardo D. (2021). Synthesis of Semi-Aromatic Di-Block Polyesters by Terpolymerization of Macrolactones, Epoxides, and Anhydrides. ChemCatChem.

[9] Van der Meulen I., Li Y., Deumens R., Joosten E.A.J., Koning C.E., Heise A. (2011). Copolymers from Unsaturated Macrolactones: Toward the Design of Cross-Linked Biodegradable Polyesters. Biomacromolecules.

[10] Witt T., Häußler M., Mecking S. (2017). No Strain, No Gain? Enzymatic Ring-Opening Polymerization of Strainless Aliphatic Macrolactones. Macromol. Rapid Commun..

[11] Tinajero-Díaz E., de Ilarduya A.M., Muñoz-Guerra S. (2020). Copolymacrolactones Grafted with l-Glutamic Acid: Synthesis, Structure, and Nanocarrier Properties. Polymer.

[12] Tinajero-Díaz E., Martínez de Ilarduya A., Muñoz-Guerra S. (2019). Synthesis and properties of diblock copolymers of ω-pentadecalactone and α-amino acids. Eur. Polym. J..

[13] Martinez A., Tlenkopatchev M.A., Gutierrez S. (2018). The Unsaturated Polyester Via Ring-Opening Metathesis Polymerization (ROMP) of *ω*-6-Hexadecenlactone. Curr. Org. Synth..

[14] Wei T., Rempel G.L., Pan Q.-M., Jiang B., Zou T.-T. (2015). A Novel Approval for Degradation of Polybutadiene and Synthesis of Diene-Based Telechelic Oligomers via Olefin Cross Metathesis. Macromol. React. Eng..

[15] Fürstner A., Langemann K. (1996). Conformationally unbiased macrocyclization reactions by ring closing metathesis. J. Org. Chem..

[16] Manzini B., Hodge P., Ben-Haida A. (2010). Entropically-driven ring-opening polymerization of macrocyclic esters with up to 84-membered rings catalysed by polymer-supported Candida antarctica lipase B. Polym. Chem..

[17] Hodge P., Colquhoun H.M. (2005). Recent work on entropically-driven ring-opening polymerizations: Some potential applications. Polym. Adv. Technol..

[18] Pepels M.P.F., Hansen M.R., Goossens H., Duchateau R. (2013). From polyethylene to polyester: Influence of ester groups on the physical properties. Macromolecules.

[19] Grubbs R.B., Grubbs R.H. (2017). 50th Anniversary Perspective: Living Polymerization—Emphasizing the Molecule in Macromolecules. Macromolecules.

[20] Yang J., Ren L., Li Y. (2017). Ring-opening metathesis polymerization of *cis*-5-norbornene-*endo*-2,3-dicarboxylic anhydride derivatives using the grubbs third generation catalyst. Chin. J. Polym. Sci..

[21] Bielawski C.W., Grubbs R.H. (2007). Living ring-opening metathesis polymerization. Prog. Polym. Sci..

[22] Lyapkov A., Kiselev S., Bozhenkova G., Kukurina O., Yusubov M., Verpoort F. (2018). Ring Opening Metathesis Polymerization. Recent Res. Polym..

[23] Yasir M., Liu P., Markwart J.C., Suraeva O., Wurm F.R., Smart M., Lattuada J., Kilbinger A.F.M. (2020). One-Step Ring Opening Metathesis Block-Like Copolymers and their Compositional Analysis by a Novel Retardation Technique. Angew. Chemie Int. Ed..

[24] Gringolts M.L., Denisova Y.I., Finkelshtein E.S., Kudryavtsev Y.V. (2019). Olefin metathesis in multiblock copolymer synthesis, Beilstein. J. Org. Chem..

[25] Fernandes H., Filgueiras J.G., de Azevedo E.R., Lima-Neto B.S. (2020). Real time monitoring by time-domain NMR of ring opening metathesis copolymerization of norbornene-based red palm olein monomer with norbornene. Eur. Polym. J..

[26] Paradiso V., Grisi F. (2019). Ruthenium-Catalyzed Alternating Ring-Opening Metathesis Copolymerization of Norborn-2-ene with Cyclic Olefins. Adv. Synth. Catal..

[27] Vasiuta R., Stockert A., Plenio H. (2018). Alternating ring-opening metathesis polymerization by Grubbs-type catalysts with: N-pentiptycenyl, N-alkyl-NHC ligands. Chem. Commun..

[28] Song A., Parker K.A., Sampson N.S. (2009). Synthesis of Copolymers by Alternating ROMP (AROMP). J. Am. Chem. Soc..

[29] Yasir M., Kilbinger A.F.M. (2021). Cascade Ring-Opening/Ring-Closing Metathesis Polymerization of a Monomer Containing a Norbornene and a Cyclohexene Ring. ACS Macro Lett..

[30] Gringolts M.L., Denisova Y.I., Shandryuk G.A., Krentsel L.B., Litmanovich A.D., Finkelshtein E.S., Kudryavtsev Y.V. (2015). Synthesis of norbornene-cyclooctene copolymers by the cross-metathesis of polynorbornene with polyoctenamer. RSC Adv..

[31] Gutekunst W.R., Hawker C.J. (2015). A General Approach to Sequence-Controlled Polymers Using Macrocyclic Ring Opening Metathesis Polymerization. J. Am. Chem. Soc..

[32] Deng L.L., Guo L.X., Lin B.P., Zhang X.Q., Sun Y., Yang H. (2016). An entropy-driven ring-opening metathesis polymerization approach towards main-chain liquid crystalline polymers. Polym. Chem..

[33] Xue Z., Mayer M.F. (2009). Entropy-driven ring-opening olefin metathesis polymerizations of macrocycles. Soft Matter..

[34] Pearce A.K., Foster J.C., O’Reilly R.K. (2019). Recent developments in entropy-driven ring-opening metathesis polymerization: Mechanistic considerations, unique functionality, and sequence control. J. Polym. Sci. Part A Polym. Chem..

[35] Yang Y., Swager T.M. (2007). Main-chain calix[4]arene elastomers by ring-opening metathesis polymerization. Macromolecules.

[36] Martínez A., Clark-Tapia R., Gutierrez S., Tlenkopatchev M. (2014). Synthesis and Characterization of New Ruthenium Vinylidene Complexes. Lett. Org. Chem..

[37] Justin R., Griffiths J.R., Diver S.T., Grubbs R.H., Wenzel A.G. (2015). Factors Affecting Initiation Rates. Handbook of Metathesis.

[38] Mayo F.R., Lewis F.M., Copolymerization I. (1944). A Basis for Comparing the Behavior of Monomers in Copolymerization; The Copolymerization of Styrene and Methyl Methacrylate. J. Am. Chem. Soc..

[39] Fineman M., Ross S.D. (1950). Linear Method for Determining Monomer Reactivity Ratios in Copolymerization. J. Polym. Sci..

[40] Erbil C., Özdemir S., Uyanik N. (2000). Determination of the monomer reactivity ratios for copolymerization of itaconic acid and acrylamide by conductometric titration method. Polymer.

[41] Schleyer P.V.R., Wiliiams J.E., Blanchard K.R. (1970). Evaluation of strain in hydrocarbons. The strain in adamantane and its origin. J. Am. Chem. Soc..

[42] Hlil A.R., Balogh J., Moncho S., Su H.L., Tuba R., Brothers E.N., Al-Hashimi M., Bazzi H.S. (2017). Ring opening metathesis polymerization (ROMP) of five- to eight-membered cyclic olefins: Computational, thermodynamic, and experimental approach. J. Polym. Sci. Part A Polym. Chem..

[43] Hodge P. (2014). Entropically driven ring-opening polymerization of strainless organic macrocycles. Chem. Rev..

